# Supplementary figures and images for: Differential Thermotolerance Adaptation between Species of Coccidioides
Source: J Fungi (Basel). 2020 Dec 14;6(4):366. doi: 10.3390/jof6040366 (PMC7765126; doi:10.3390/jof6040366)

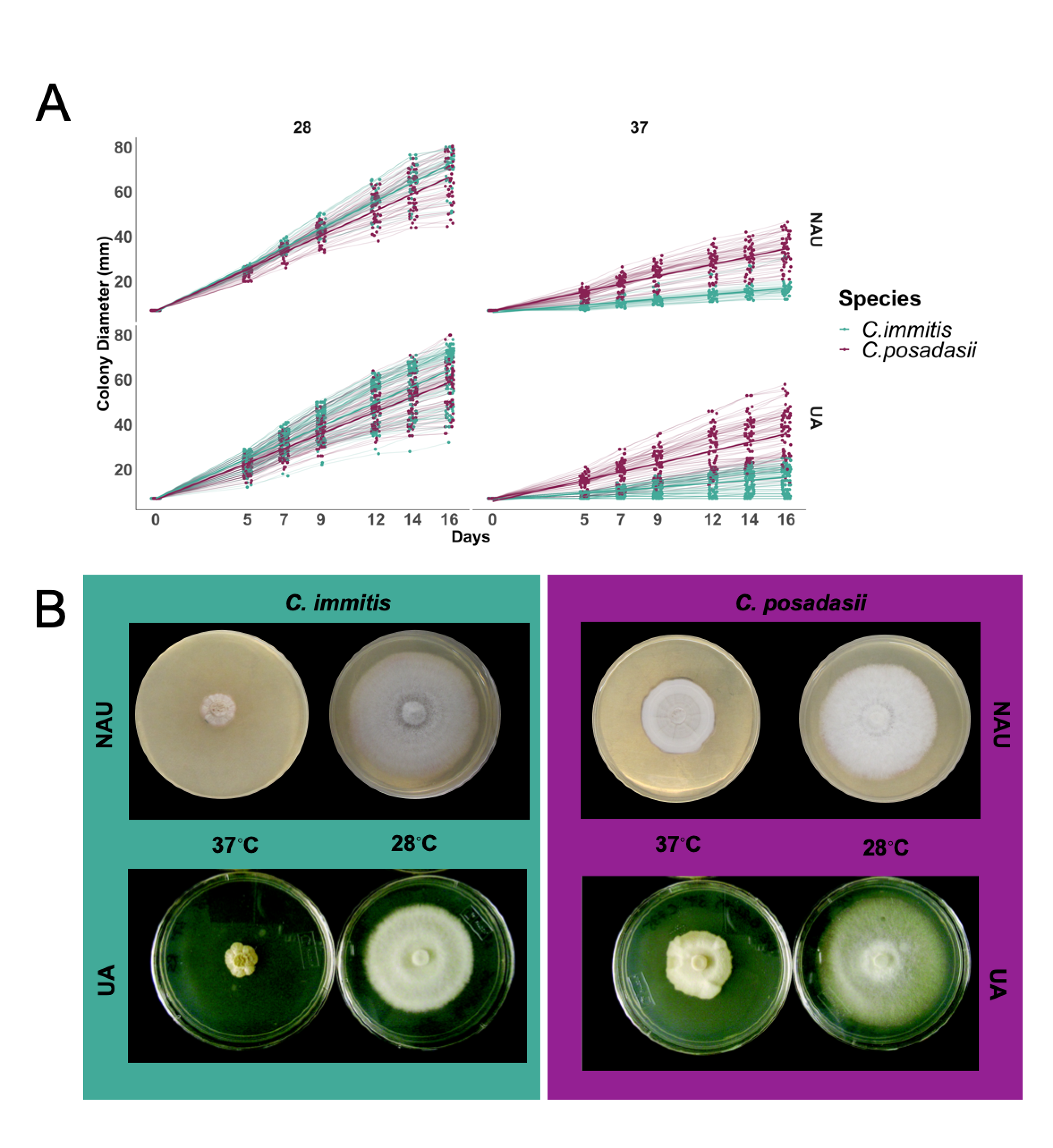

Supplement: Supplementary file 1 [file jof-06-00366-s001.zip › supplemental data/S1Fig.tiff]
