# Supplementary material for: Differential Thermotolerance Adaptation between Species of Coccidioides
Source: J Fungi (Basel). 2020 Dec 14;6(4):366. doi: 10.3390/jof6040366 (PMC7765126; doi:10.3390/jof6040366)
Supplement: Supplementary file 1 [file jof-06-00366-s001.zip › supplemental data/Table S1.doc]

| **Supplemental Table 1.**  **Type III Analysis of Variance Table with Satterthwaite's method** | | | | | | |  |
| --- | --- | --- | --- | --- | --- | --- | --- |
| *Factor* | *Sum Sq* | *Mean Sq* | *NumDF* | *DenDF* | *F value* | *Pr(>F)* | |
| 28ºC Specific model. |  |  |  |  |  |  | |
| Species | 6407.45 | 3203.72 | 2 | 1707.19 | 449.80 | 0.00 | |
| Day | 18533.00 | 18533.00 | 1 | 86.07 | 2602.03 | 0.00 | |
| Lab | 1.42 | 1.42 | 1 | 1707.19 | 0.20 | 0.66 | |
| Species:Day | 78.86 | 78.86 | 1 | 86.07 | 11.07 | 0.00 | |
| Species:Lab | 26.78 | 26.78 | 1 | 1707.19 | 3.76 | 0.05 | |
| Day:Lab | 358.12 | 358.12 | 1 | 106.00 | 50.28 | 0.00 | |
| Species:Day:Lab | 69.81 | 69.81 | 1 | 106.00 | 9.80 | 0.00 | |
| 37ºC Specific Model |  |  |  |  |  |  | |
| Species | 6084.08 | 3042.04 | 2 | 1714.46 | 1493.34 | 0.00 | |
| Day | 1101.08 | 1101.08 | 1 | 83.44 | 540.52 | 0.00 | |
| Lab | 24.06 | 24.06 | 1 | 1714.46 | 11.81 | 0.00 | |
| Species:Day | 173.42 | 173.42 | 1 | 83.44 | 85.13 | 0.00 | |
| Species:Lab | 24.97 | 24.97 | 1 | 1714.46 | 12.26 | 0.00 | |
| Day:Lab | 15.00 | 15.00 | 1 | 93.96 | 7.36 | 0.01 | |
| Species:Day:Lab | 39.13 | 39.13 | 1 | 93.96 | 19.21 | 0.00 | |

Analysis of variance. Impact of institution collection site was investigated for each temperature specific model. The factors “day”, “species”, and “lab” were assumed to be fixed linear effects, and individual isolate response for each day was considered to be a normally distributed random effect as appropriate in a longitudinal study. The Factor “Lab” location adds variation to the data sets but does not alter overall findings. Species specific behavior based on temperature pattern remain.
